# Impact of Mean Platelet Volume on Long-Term Mortality in Chinese Patients with ST-Elevation Myocardial Infarction

**DOI:** 10.1038/srep21350

**Published:** 2016-02-16

**Authors:** Xi-peng Sun, Bo-yu Li, Jing Li, Wei-wei Zhu, Qi Hua

**Affiliations:** 1 Department of Cardiology, Xuanwu Hospital, Capital Medical University, Beijing, China, 100053

## Abstract

We investigated the association between mean platelet volume (MPV) and risk of all-cause mortality in Chinese patients with ST-Elevation Myocardial Infarction (STEMI). We enrolled 1836 patients with STEMI in Xuanwu Hospital from January 2008 to December 2013. Based on MPV, patients were categorized into the following groups: <9.5 fL (n = 85), 9.5–11.0 fL (n = 776), 11.1–12.5 fL (n = 811) and >12.5 fL (n = 164), respectively. Mean duration of follow-up was 56.9 months, and 197 patients (10.7%) died during follow-up. All-cause mortality rates were compared between groups. The lowest mortality occurred in patients with MPV between 9.5–11.0 fL, with a multivariable-adjusted hazard ratio (HR) of 1.15(95%CI 0.62–1.50), 1.38(95%CI 1.20–1.68), and 1.72(95%CI 1.41–1.96) in patients with MPV of <.5, 11.1–12.5 and >12.5 fL, respectively. Therefore, increased MPV was associated with all-cause mortality in Chinese patients with STEMI. MPV might be useful as a marker for risk stratification in Chinese patients with STEMI.

ST-Elevation Myocardial Infarction (STEMI) is a major public health problem and a leading cause of death both in developed and developing countries. The morbidity and mortality of patients with STEMI remain high despite current advances in drug therapy and revascularization techniques such as percutaneous coronary intervention (PCI) and coronary artery bypass graft surgery (CABG). Considering the poor prognosis, useful biomarkers for predicting future cardiovascular events are needed. Circulating platelets are heterogeneous in size, density and reactivity, and changes in these variables are important for the development of acute coronary syndromes (ACS). Mean platelet volume (MPV), a measurement of the variability in size of circulating platelets, has been previously suggested to be an important hematologic variable and indicator of platelet function^1^.

According to present studies, increased MPV may be associated with poor clinical outcome among survivors of ACS^2^,^3^,^4^,^5^,^6^,^7^,^8^. Although the underlying biological mechanisms remain unclear, several potential factors have been postulated, such as larger platelets are metabolically and enzymatically more active than smaller platelets. Larger platelets have a greater content of granules, increased thromboxane synthesis, β-thromboglobulin, serotonin release, increased expression of P-selectin, glycoprotein IIb/IIIa and fibrinogen receptors^9^,^10^,^11^,^12^.

In the present study, we used a retrospective database to investigate the relation between MPV and risk of all-cause mortality in Chinese patients with STEMI.

## Results

### Baseline Characteristics

1836 patients with STEMI were enrolled in Xuanwu Hospital from January 2008 to December 2013, and categorized into the following four groups: <9.5 fL (n = 85), 9.5–11.0 fL (n = 776), 11.1–12.5 fL (n = 811) and >12.5 fL (n = 164), respectively. Baseline characteristics and treatments were shown in Table 1. Patients with higher MPV were more likely to be older, and had higher triglyceride (TG), low density lipoprotein cholesterol (LDL-C), white blood cell (WBC) and prevalence of prior myocardial infarction, and had reduced platelet levels. No significant differences in coronary revascularization and medications were identified between the groups.

In a multivariable linear regression model, MPV were associated with age, TG, LDL-C, WBC, platelet and prior myocardial infarction (Table 2).

### Clinical Outcomes

**Figure 1:**
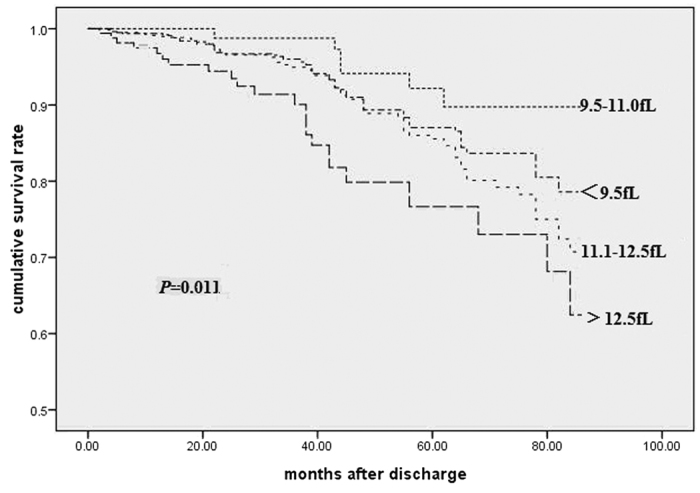
Kaplan-Meier Curves of Cumulative Survival Rate.

Patients were followed between 2 and 7 years (mean 56.9months). 197 patients (10.7%) died, including 144 deaths from cardiac causes (recurrent myocardial infarction, n = 41; heart failure, n = 75; serious cardiac arrhythmias, n = 23; and sudden death, n = 5) during the follow-up period. Kaplan-Meier curves showed that the lowest cumulative survival rate occurred in patients with MPV >12.5 fL, while the lowest mortality occurred in patients with MPV between 9.5–11.0 fL (*p* = 0.011; Fig. 1). The results of the Cox proportional hazards model examining the relationship between MPV and all-cause mortality using 9.5–11.0 fL as the reference group. After adjustment for other factors independently associated with mortality, the risk associated with lower MPV was no longer significant. The risk associated with elevated MPV was attenuated but remained statistically significant with increased risk for mortality even for patients with MPV within the normal range (Table 3).

## Discussion

The main finding in the present study was that increased MPV was associated with all-cause mortality in Chinese patients with STEMI. The association remained significant even after adjustment for other independent variables.

Platelets are quite heterogeneous blood elements, diverging in terms of size, density and reactivity. The changing of these parameters might be associated with various diseases either as a triggering or propagating factor. In general, the smaller platelet size indicates low production state such as aplastic anemia or myelodystrophic states^13^, while the larger size indicates a higher destruction rate such as autoimmune related or other acute inflammatory states^14^. MPV, which is the most accurate measure of the size of platelets, is a simple and easy measurement. A higher MPV has been previously observed in patients with a history of smoking^15^, diabetes mellitus^16^, cerebrovascular disease^17^,^18^, congestive heart failure^19^ and in hypertensive patients with evidence of target organ damage^20^.

Recent studies have showed that a higher MPV might be identified as a marker of increased morbidity and mortality in patients with ACS, especially in STEMI. Martin *et al*. found that higher MPV values were associated with increased subsequent ischemic events among patients with acute myocardial infarction^21^. Huczek *et al*.demonstrated MPV was associated with the no-reflow phenomenon and clinical outcome in patients with STEMI^2^. Along these lines, Maden *et al.* reported that MPV was correlated with the presence of infarct-related artery patency (IRA) in a cohort of 351 STEMI patients^22^. Sezer *et al.* demonstrated a relationship between MPV values and coronary microvascular injury in patients undergoing PCI^23^. Rodrigo *et al.* thought that MPV could predict patency of the IRA before mechanical reperfusion and short-term mortality in patients with STEMI undergoing PCI^24^. Sarli *et al.* reckoned that MPV was associated with poor post interventional myocardial blush grade in patients with STEMI^6^. On the other hand, Yang *et al.* showed that MPV was a marker of restenosis after percutaneous transluminal coronary angioplasty (PTCA) in patients with stable and unstable angina pectoris^25^. Additionally, Acar *et al.* found an association between higher MPV and impaired function of the left ventricle after percutaneous revascularization of the coronaries^26^. Similarly, we also found that increased MPV was associated with long-term mortality in Chinese patients with STEMI, even after adjustment of other factors.

Although recent studies have showed that increased MPV was an independent risk factor for poor outcomes in cardiovascular disease, the mechanistic links between MPV and poor prognosis in cardiovascular disease have not yet been fully understood. Thrombus formation in the setting of acute myocardial infarction is a complex dynamic process involving both thrombogenesis and thrombolysis and platelets are of pathogenic importance in coronary atherosclerosis and its complications. It has been shown that larger platelets are metabolically and enzymatically more active than smaller ones and aggregate easily^27^. They express higher levels of prothrombotic substance, thromboxan A_2_, serotonin, β-thromboglobulin, and procoagulatory surface proteins such as P-selectin and glycoprotein IIIa. These inflammatory makers play a pathogenic role in atherogenesis and cardiovascular disease progression and instability^28^. An increased MPV decreases the inhibitory effectiveness of prostacyclin I2 (PGI2) on both platelet aggregation and the release reaction^29^. A shortening of bleeding time and a higher level of P-selectin were previously reported to associate with acute myocardial infarction. Cytokines may possibly trigger the production of larger, more reactive platelets following platelet destruction in peripheral blood. Moreover, elevated levels of CD40 ligands, which are expressed by activated platelets, have been found in atheromatous plaques^30^. Based on the above Analysis, further studies are needed to clarify the relationship between MPV and different clinical endpoints.

Some limitations in the present study must be considered. This was a single-center, retrospective analysis. Residual confounding factors might thus have affected the results, regardless of the adjusted analysis. Further studies are needed to clarify the mechanisms underlying the relationship between MPV and mortality.

In conclusion, increased MPV was associated with all-cause mortality in Chinese patients with STEMI. MPV might be useful as a marker for risk stratification in Chinese patients with STEMI.

## Methods

### Study Population

This was a single-center, retrospective analysis. This retrospective study was approved by the Ethics Committee of Xuanwu Hospital, Capital Medical University. The methods were carried out in accordance with the approved guidelines. Since this was a retrospective study, the requirement for informed consent was waived. We enrolled 1836 patients with STEMI in Xuanwu Hospital from January 2008 to December 2013. STEMI was a clinical syndrome defined by characteristic symptoms of myocardial ischemia in association with persistent electrocardiographic (ECG) ST elevation and subsequent release of biomarkers of myocardial necrosis. We excluded patients with any blood, renal, hepatic, autoimmune diseases, malignancy, and the use of thrombolytic drugs within the previous 24 hours. Baseline blood analyses were measured on admission. Demographic data (including age, gender and so on), treatments and comorbidities (hypertension, diabetes, dyslipidemia, prior myocardial infarction and cerebrovascular disease) were also recorded.

### Classification of MPV

Baseline MPV was measured on admission using XE-5000 automated hematology analyzer (Sysmex, Kobe, Japan) and the normal range was 9.5–12.5 fL. The majority of patients (n = 1587, 86.5%) had MPV within the normal range, while 85 (4.6%) patients below normal range and 164 (8.9%) above normal range. Considering the small numbers of patients with MPV out of the normal range, the relation between MPV (if it’s divided by tertiles or quartiles) and clinical outcome would be attenuated. It’s more significant when MPV was divided by reference range. Thus, to evaluate the relation between MPV and clinical outcome, the 1836 patients were categorized into the following groups: <9.5 fL (n = 85), 9.5–11.0 fL (n = 776), 11.1–12.5 fL (n = 811) and >12.5 fL(n = 164), respectively.

### Clinical Outcome

The end-point of this study was the incidence of all-cause mortality. Cardiac death was defined as death caused by recurrent myocardial infarction, heart failure, serious cardiac arrhythmias and sudden death. Clinical outcome data were collected until 1st November 2015 and mean duration of follow-up was 56.9 months after hospital discharge. Clinical event data were fully collected during the follow-up period for all patients by reviewing the national death registry and by contacting each patient individually and independently reviewing the hospital course for major clinical events if the patient had been rehospitalized.

### Statistical Analysis

For baseline characteristics, variables were summarized as percentages for discrete variables and means ± standard deviations for continuous variables. We used χ^2^ test or analysis of variance, respectively, to test for differences in categorical or continuous factors between different groups of MPV.

Multivariate linear regression was used to determine factors associated with MPV. We used Cox proportional-hazards model with backward selection to examine the association between MPV and clinical outcome. Variables considered for inclusion in the multivariable model included: age, gender, TG, LDL-C, WBC, platelet, coronary revascularization, medication, and history of hypertension, diabetes mellitus, dyslipidemia, prior myocardial infarction and cerebrovascular disease. Survival curves were constructed using the Kaplan–Meier method, and comparisons were made using the log–rank test. Statistical analyses were performed using the SPSS statistical software version 17.0 (Chicago, IL). Differences were considered statistically significant at the 2-sided *P* < 0.05 level.

## Additional Information

**How to cite this article**: Sun, X.-p. *et al.* Impact of Mean Platelet Volume on Long-Term Mortality in Chinese Patients with ST-Elevation Myocardial Infarction. *Sci. Rep.* **6**, 21350; doi: 10.1038/srep21350 (2016).

## Figures and Tables

**Table 1:** Baseline Characteristics.

	MPV(fL)				*P* value
	<9.5 (n = 85)	9.5–11.0 (n = 776)	11.1–12.5 (n = 811)	>12.5 (n = 164)	
Age (years)	59.1 ± 13.2	60.7 ± 10.9	61.4 ± 9.6	62.5 ± 12.7	0.006
Female, n (%)	23 (27.4)	227 (29.3)	228(28.1)	47(28.8)	0.075
Hypertension, n (%)	50 (58.9)	452 (58.2)	462 (57.0)	99 (60.4)	0.094
Diabetes mellitus, n (%)	23 (26.7)	213 (27.5)	232 (28.6)	44 (27.1)	0.13
Dyslipidemia, n (%)	47 (55.4)	436 (56.2)	448 (55.3)	95 (57.8)	0.27
Prior myocardial infarction, n (%)	5 (6.1)	64 (8.2)	61 (7.5)	15 (8.9)	0.018
Cerebrovascular disease, n (%)	11 (13.2)	119 (15.3)	117 (14.4)	24 (14.8)	0.16
Cr (mg/dL)	0.85 ± 9.5	0.84 ± 11.4	0.86 ± 10.3	0.86 ± 11.2	0.46
BUN (mg/dL)	13.88 ± 1.2	13.44 ± 1.9	14.08 ± 1.5	13.76 ± 1.5	0.38
ALT (U/L)	22.3 ± 10.7	21.5 ± 12.4	21.6 ± 11.5	23.7 ± 13.3	0.14
AST (U/L)	24.3 ± 11.8	24.8 ± 14.2	25.1 ± 10.5	23.7 ± 11.2	0.26
T-Cho (mg/dL)	166.4 ± 1.0	170.2 ± 1.3	174.9 ± 1.2	171.0 ± 1.1	0.53
TG (mg/dL)	163.2 ± 1.2	162.4 ± 1.5	169.3 ± 1.1	181.8 ± 1.1	0.019
HDL-C (mg/dL)	36.7 ± 0.2	39.6 ± 0.3	40.1 ± 0.3	41.0 ± 0.3	0.43
LDL-C (mg/dL)	92.5 ± 0.8	93.6 ± 0.7	94.1 ± 0.6	98.7 ± 0.7	0.047
Fasting plasma glucose (mg/dL)	103.4 ± 2.1	106.5 ± 2.3	105.2 ± 1.8	103.9 ± 2.1	0.85
WBC ( × 10^9^/L)	5.3 ± 2.4	6.4 ± 2.1	6.7 ± 1.9	7.8 ± 2.0	0.032
Hemoglobin (g/dL)	13.5 ± 14.9	13.9 ± 17.2	13.8 ± 14.3	13.1 ± 18.3	0.94
Platelet ( × 10^9^/L)	254.3 ± 72.2	231.9 ± 63.9	216.1 ± 65.8	201.7 ± 69.8	0.012
Coronary revascularization, n (%)	70 (82.4)	653 (84.1)	685 (84.5)	138 (83.9)	0.092
Aspirin, n (%)	83 (97.8)	752(96.9)	776 (95.7)	159 (97.2)	0.66
DAPT, n (%)	78 (92.1)	725 (93.4)	746(92.0)	152 (92.7)	0.46
Statin, n (%)	72 (84.2)	652 (84.0)	687 (84.7)	140 (85.1)	0.73
β-blocker, n (%)	55 (64.2)	493 (63.5)	521 (64.3)	103 (62.9)	0.62
Ca-blocker, n (%)	36 (41.8)	320 (41.2)	345 (42.5)	71 (43.1)	0.39
ACE-I/ARB, n (%)	54 (63.4)	485 (62.5)	503 (62.0)	104 (63.2)	0.58
OHA, n (%)	21 (24.5)	199 (25.6)	200 (24.7)	41 (25.0)	0.72
Insulin, n (%)	15 (17.4)	131 (16.9)	135 (16.6)	29 (17.6)	0.87

MPV: mean platelet volume; Cr: creatinine; BUN: blood urea nitrogen; ALT: glutamic-pyruvic transaminas; AST: glutamic-oxaloacetic transaminase; T-Cho: total cholesterol; TG: Triglyceride; HDL-C: high density lipoprotein cholesterol; LDL-C: low density lipoprotein cholesterol; WBC: white blood cell; DAPT: dual antiplatelet therapy; ACE-I: angiotensin-converting enzyme inhibitor; ARB: angiotensin receptor blocker; OHA: oral hypoglycemic agents.

**Table 2:** Factors Associated with MPV in Multivariable Linear Regression.

Independent Variable	Regression Coefficient (SE)	95% CI	P value
Age(per decade)	0.10(0.02)	0.06 to 0.22	<0.001
Prior myocardial infarction	0.08(0.02)	0.08 to 0.16	0.003
TG	0.08(0.03)	0.07 to 0.14	0.005
LDL-C	0.05(0.05)	0.08 to 0.15	0.003
WBC	0.04(0.02)	0.06 to 0.12	0.008
Platelet	−0.06 (0.04)	−0.12 to -0.04	<0.001

SE: standard error; 95%CI: 95% confidence interval; TG: Triglyceride; LDL-C: low density lipoprotein cholesterol; WBC: white blood cell.

**Table 3:** Adjusted Association between MPV and Clinical Outcome.

	Events, n (%)	Unadjusted			Adjusted		
		HR (95% CI)	*P* value	*P*trend	HR (95% CI)	*P* value	*P*trend
<9.5fL	9(10.6)	1.22(0.73–1.53)	0.07		1.15(0.62–1.50)	0.24	
9.5–11.0fL	72(9.3)	1.0 (Referent)	–	<0.001	1.0 (Referent)	–	0.002
11.1–12.5fL	92(11.3)	1.45 (1.28–1.77)	0.004		1.38 (1.20–1.68)	0.007	
>12.5fL	24(14.6)	1.81 (1.48–2.06)	<0.001		1.72(1.41–1.96)	0.004	

HR: hazard ratio; 95%CI: 95% confidence interval.
